# In vitro and in vivo double-enhanced suicide gene therapy mediated by generation 5 polyamidoamine dendrimers for PC-3 cell line

**DOI:** 10.1186/1477-7819-10-3

**Published:** 2012-01-08

**Authors:** Yue Chen, Gang Wang, Deling Kong, Zhihong Zhang, Kuo Yang, Ranlu Liu, Weiming Zhao, Yong Xu

**Affiliations:** 1 Department of Urology, Second Hospital of TianJin Medical University, TianJin Institute of Urology, Tianjin, China; 2 Department of Urology, HeBei General Hospital, Shijiazhuang, HeBei province, China; 3 The Key Laboratory of Bioactive Materials, Ministry of Education, College of Life Science, Nankai University, Tianjin, China; 4 Department of Urology, The First Affiliated Hospital of Harbin Medical University, Harbin, China

## Abstract

**Background:**

One of the most frequently used and efficient suicide gene therapies for prostate cancer is HSV-TK/GCV system, but its application has been limited due to lack of favorable gene vector and the reduction of "bystander effect". We investigated the effect of a novel combination of HSV-TK/GCV fused with Cx43 and gemcitabine using non-viral vector generation 5 polyamidoamine dendrimers (G5-PAMAM-D) on PC-3 cells.

**Methods:**

RT-PCR and Western blot were used to detect TK and Cx43 expression. Cell viability and proliferation were measured by using MTT assay. Cell apoptosis was detected with double-staining of Annexin V-FITC and propidium iodide (PI) by flow cytometry. Nude mice models were established to evaluate the therapeutic effect in vivo.

**Results:**

G5-PAMAM-D efficiently delivered recombinant plasmids into PC-3 cells and HSV-TK and Cx43 could be expressed successfully. With gemcitabine, G5-PAMAM-D mediated HSV-TK and Cx43 expression effectively inhibited prostate cancer PC-3 cell proliferation, leading to more cellular apoptosis and inhibiting PC-3 tumor growth in nude mice models.

**Conclusions:**

This study illustrates that this new suicide gene system mediated by G5-PAMAM-D is effective in decreasing PC-3 cell proliferation and inducing cell apoptosis, and inhibiting tumor growth in vivo. In a word, our study could provide a potential approach for gene therapy of prostate cancer.

## Background

The incidence and mortality of prostate cancer are gradually increasing in the world in recent years [1]. Localized prostate cancer can be managed effectively with surgery or radiation [2]. However, tumor progression is eventually inevitable after hormonal deprivation therapy for most patients [3]. There are no curable approaches for prostate cancer in an androgen independent state [4]. For this reason, it is necessary to search for new and effective therapy. Gene therapy is a hopeful therapeutic approach for prostate cancer [5]. While suicide gene therapy is an attractive approach, in which a gene encoding for a metabolic enzyme that can convert a nontoxic prodrug into a toxic compound is introducted into tumor cells. The nontoxic prodrug can be given at high doses to maintain appropriate treatment in tumor cells, without negative effects to the normal cells. It is considered that suicide gene therapy is the focus of the cancer gene therapy research because of its "bystander effect" [6]. The "bystander effect" is a phenomenon that the transduction of a small fraction of tumor cells with the suicide gene can result in widespread tumor cell death. It can compensate for the lowered efficacy of vectors and accomplish complete killing of a tumor. The most frequently used suicide gene therapy for prostate cancer is Herpes Simplex Virus Thymidine Kinase (HSV-TK)/ganciclovir (GCV) system [7,8]. However, the reduction of "bystander effect" and the safety problems of viral vectors need to be solved urgently.

Adenovirus vectors are the most common viral vectors used for anti-cancer therapy in human clinical studies [9]. Although adenoviral vectors have demonstrated high gene transfer efficiency, the major disadvantage of these vectors is immunogenicity. That is to say, immune responses probably induced by the repeat application of adenoviral vectors may result in the removal of vectors in human body. Generation 5 polyamidoamine dendrimers (G5-PAMAM-D) is a kind of new synthetic nanoparticles polymer material. Compared with viral vectors, its advantages lie within its non-toxic and non-immunogenic nature, in addition to its capability to carry larger pieces of DNA.

Study has indicated that "bystander effect" is essential in suicide gene therapy [10]. Increasing the density of gap junctions can enhance the "bystander effect" by expression of connexin 43 (Cx43) [11]. When gemcitabine were used for HSV-TK/GCV suicide gene therapy of glioma, the "bystander effect" was significantly enhanced [12]. In this study, we will explore the effect of expression of Cx43 and gemcitabine in suicide gene therapy for prostate cancer.

## Methods

### Cell culture and animals

Human prostate cancer PC-3 cells were obtained from our TianJin Institute of Urology. PC-3 cells were maintained at 37°C in a humidified atmosphere of 5% CO_2 _in RPMI1640 medium (Gibco, USA), supplemented with 10% fetal bovine serum (FBS) (Gibco, Uruguay), penicillin (100 U/ml) and streptomycin (100 μg/ml). Six-week-old male BALB/c nude mice (Animal Resources Centre, Military Medical Sciences, BeiJing, China) were used for the experiments. Mice were maintained under specific pathogen-free, temperature-controlled conditions at the animal facilities of College of Life Science in Naikai University. Mice were handled in accordance with Institutional Animal Welfare Guidelines.

### Vectors and plasmids

G5-PAMAM-D is synthesized and kindly provided by Key Laboratory of Bioactive Materials of Ministry of Education of Nankai University [13]. Plasmid pCMV-TK-Cx43 and pCMV-TK had been successfully constructed in our laboratory. Cx43 gene was amplified by PCR from HL60 genomic DNA and subcloned into pMD19-T Simple vector, which was digested by Sal I and Not I, named pMD-Cx43. TK gene was synthesized from pORF-HSV-TK (TakaRa, China) and was inserted into pMD19-T Simple vector with Xho I and Mlu I, producing pMD-TK. Then the pIRES and pMD-TK plasmids were degested by Xho I and Mlu I and TK fragment was cloned into multiple clone site (MCS) A of pIRES to generate the pCMV-TK. The pCMV-TK and pMD-Cx43 plasmids were digested by Sal I and Not I. Then the Cx43 fragment was inserted into MCS B of pCMV-TK plasmid. The new plasmid was named pCMV-TK-Cx43. Escherichia coli strain DH5a carrying recombinant plasmids were shaking cultured in LB medium (250 ml) containing ampicillin (100 μg/ml) at 37°C for 16-18 h. Two plasmids were extracted using Plasmid Purification Maxi Kits (QIAGEN, China).

### Cell transfection

PC-3 cells were plated until they reached 70-80% confluency, then different mixtures (G5-PAMAM-D mixed with two different plasmids at a 1:3 molar ratio respectively) were added gently to cells covered with serum-free medium. All the cells were divided into 7 groups: Group A, B (G5-PAMAM-D/pCMV-TK-Cx43); Group C, D (G5-PAMAM-D/pCMV-TK); Group E (G5-PAMAM-D); Group F (pCMV-TK-Cx43); Group G (neither vector nor plasmid). Gemcitabine would be added to group A, C and F when MTT assay and apoptosis detection were conducted. After incubation for 6 h at 37°C, the medium was replaced with fresh serum-containing medium. After 48 h of incubation, cells were used for different experiments.

### RT-PCR analysis

Total cell RNAs were isolated 48 h after transfection using Multisource Total RNA Miniprep Kit (AXYGEN, China). The synthesis of cDNA and PCR were performed using one-step RT-PCR system (TransGen Biotech Co. Ltd, Beijing, China). PCR was run in a 50 μl reaction volume. The PCR specific primer pairs (TaKaRa, China) were 5'-CCGCTCGAGATGGCCTCGTACCCCGGCCATCAACA-3'(forward) and 5'-CGAAAGCTTACCAGAACCACCGTTAGCCTCCCCCATCTCCCGGGCA-3' (reverse) for analysis of TK, 5'-GTCGACATGGGTGACTGG AGCGCCTT-3' (forward) and 5'-GCGGCCGCCTAGATCTCCAGGTCATCAGG-3'(reverse) for analysis of Cx43. The amplified product of TK had 1143 bp and that of Cx43 had 1149 bp. The PCR products of TK and Cx43 were analyzed by 0.8% agarose gel electrophoresis.

### Western blot analysis

48 h after transfection, cells were rinsed twice with cold PBS, then homogenized in RIPA cell lysate and centrifuged. The protein concentration of each sample was quantified by the Bradford assay. Fifty micrograms of total cell extract protein was separated by 10% sodium dodecyl sulfate-polyacrylamide gel electrophoresis (SDS-PAGE) and transferred onto polyvinylidene difluoride (PVDF) membrane. After being blocked with 5% skimmed milk for 1 h at room temperature, the membrane was incubated with goat anti-TK (Santa Cruz Biotechnology, CA, USA) or rabbit anti-Cx43 (Abcam, Cambridge, MA) antibody overnight at 4°C. After extensive washes, blots were incubated with the dilution (1:2000) horseradish peroxidase conjugated anti-goat or anti-rabbit IgG (Santa Cruz Biotechnology, CA, USA) for 90 minutes at 37°C. The bands were developed with 3,3'-diaminobenzidine (DAB). GAPDH (Santa Cruz Biotechnology, CA, USA) was used as an internal control.

### Cell proliferation assay

3-(4,5-dimethylthiazol-2-yl)-2,5-diphenyl-tetrazoliumbromide (MTT), GCV, gemcitabine were all purchased from Sigma (USA). 48 h after transfection, 200 μl of the complete medium was replaced with that containing different concentrations of GCV (0.1, 1, 5, 0, 20, 50, 100, 200, 500 μg/ml) and gemcitabine (500 μmol/l) was added to Group A, C and F at the same time. After incubation for 6 h (in 5% CO_2 _at 37°C), fresh complete medium was replaced. Then 20 μl of 5 mg/ml MTT solution was added to each well. After 4 h of incubation at 37°C, the supernatant was removed and 150 μl of DMSO were added to each well. The absorbance of optical density at 570 nm (A_570_) was measured using a Microplate Reader. Cell growth inhibition rate was calculated according to the formula (A_570 _of control cells-A_570 _of treated cells) × 100%/A_570 _of control cells and the curve was drawn.

### Detection of cell apoptosis

The detection of cell apoptosis was performed according to the instructions of Annexin V-FITC apoptosis kit (Beckman Coulter, CA, USA). 48 h after transfection, 200 μl of the complete medium containing GCV (50 μg/ml) was administrated and gemcitabine (500 μmol/l) was added to Group A, C and F at the same time. After incubation for 6 h, cells were centrifuged and resuspended with cold binding buffer at concentration 1.0 × 10^6^/ml. 100 μl of the cell suspension was added to 5 ml flow pipe mixed with 5 μl Annexin V-FITC and 10 μl PI. After dark incubation for 15 minutes at room temperature, flow cytometry was used to detect apoptotic cells.

### In vivo antitumoral activity

A suspension of PC-3 cells (4 × 10^6 ^cells) in 200 μl of mixture containing PBS and Matrigel matrix basement memberane (BD Corporation, USA) at 3:1 ratio of volume was injected subcutaneously into the right armpit regions of mice. When the tumor volume reached approximately 0.1 cm^3^, usually ten days after cell implantation, the mice were randomly divided into eight groups (five animals per group). The mice were submitted to intratumoral injection three times (day1, 7 and 13) with the mixture of G5-PAMAM-D/pCMV-TK-Cx43(Group I and II), the mixture of G5-PAMAM-D/pCMV-TK(Group III and IV), the mixture of G5-PAMAM-D (Group V), the mixture of pCMV-TK-Cx43 (Group VI), PBS (Group VII) and no treatment (Group VIII). 24 hours after the injections of mixtures every time, GCV (100 mg/kg) was administered intraperitonial once a day for 5 consecutive days. Gemcitabine (20 mg/kg) was administrated intraperitonial in Group I, III and VI at day 2, 8 and 14 respectively. The tumor volume was calculated at regular intervals according to the formula V = π/6 × length × width^2^. After mice were sacrificed, tumors were extracted and weighted. Tumor specimens were used for histological analysis.

### Statistical analysis

Statistical analysis was performed using SPSS software (version 13.0). The significance of differences between multiple groups was determined by one-way analysis of variance followed by the LSD test. Data was expressed as means ± standard deviation. Statistical significance was set at P < 0.05.

## Results

### Expressions of TK and Cx43 after PC-3 cells transfection

**Figure 1:**
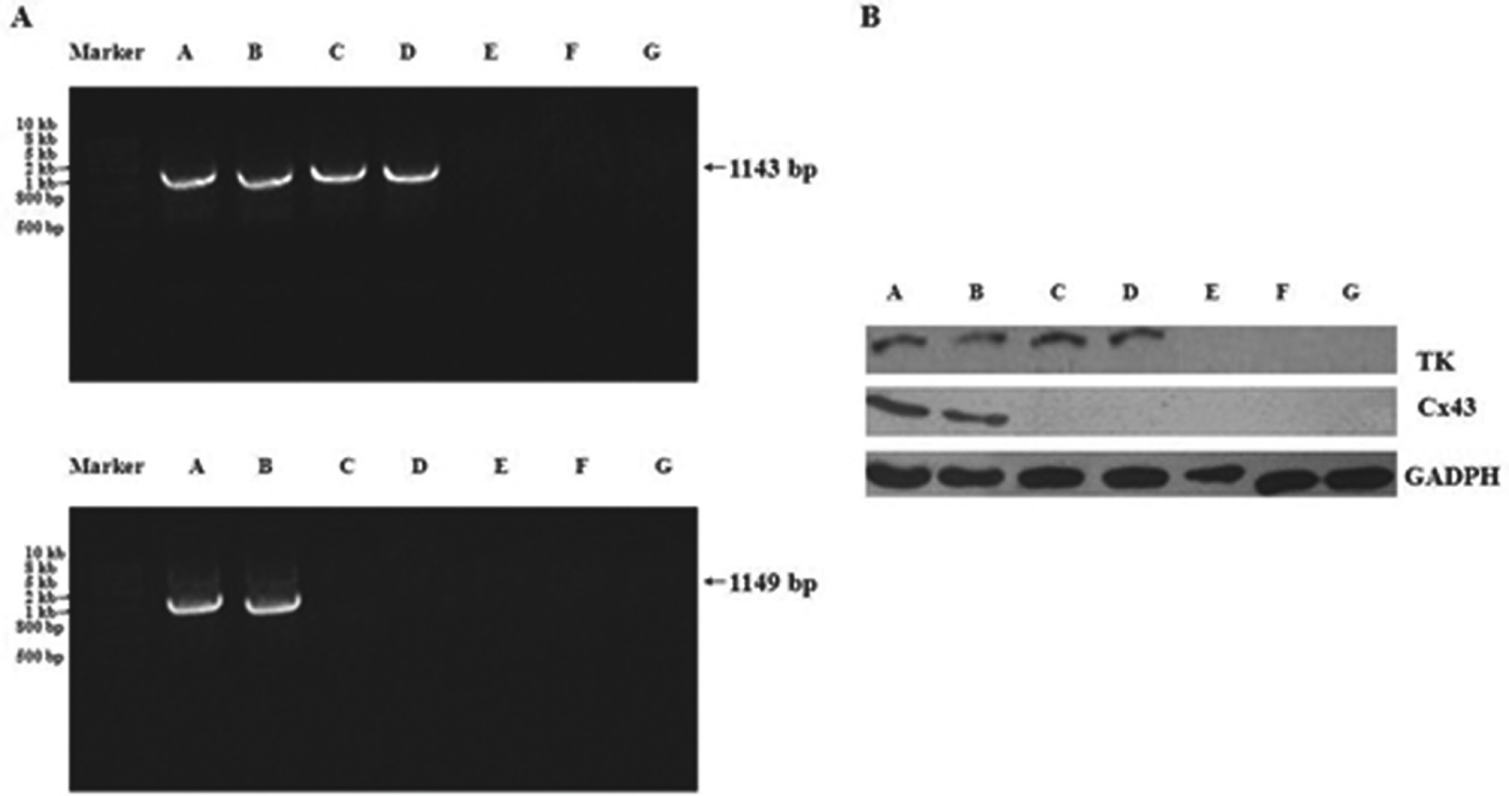
**Expressions of TK and Cx43 after PC-3 cells transfection**. **A**. The TK bands of 1143 bp were detected in Group A, B, C, D and the Cx43 bands of 1149 bp were only detected in Group A and B. **B**. The TK proteins were detected in Group A, B, C, D and the Cx43 proteins were only detected in Group A and B. Equal loading was confirmed by stripping the blot and reprobing it for GAPDH.

To evaluate whether recombinant plasmids can be successfully transferred into PC-3 cells by using G5-PAMAM-D, and whether TK and Cx43 genes can be expressed, total PC-3 cell RNAs and cell proteins were extracted from different groups after transfection. The expressions of TK and Cx43 were detected by RT-PCR and Western blot. As shown in Figure 1, TK was expressed in Group A to D and Cx43 was expressed in Group A and B. Neither TK nor Cx43 was expressed in Group E to G.

### Double-enhanced suicide gene system suppresses PC-3 cell growth

**Figure 2:**
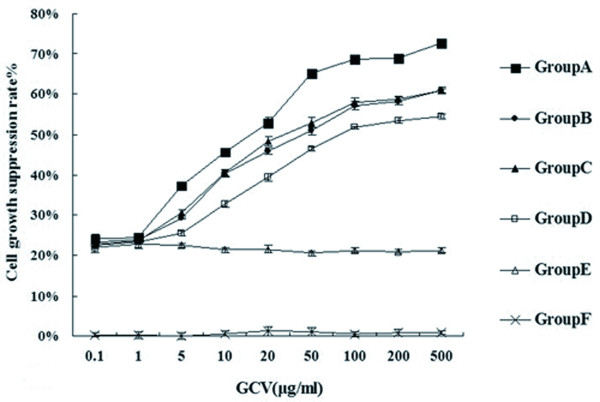
**Cell growth inhibition rate curve**. Cell growth inhibition rate curves were drawn to evaluate cell growth according to MTT assay. The results showed different growth inhibition rates of PC-3 cells in all groups. The Group A had an obviously higher growth inhibition rate than other groups (P < 0.01).

To investigate the effect induced by this new suicide gene system, the level of cell proliferation was assessed by the MTT assay. As shown in the cell growth inhibition rate curve (Figure 2), cell growth inhibition rates of Group A to D gradually increased in different extent with the increasing concentrations of GCV, and the cell growth inhibition rate of Group A was much higher than other groups under the same experimental conditions (P < 0.01). Cell proliferations almost were not influenced in Group F at different GCV concentrations. In addition, the cell growth inhibition rate of Group E stayed at much lower level than those of Group A to D (P < 0.01).

### Double-enhanced suicide gene system induces apoptosis in PC-3 cells

**Figure 3:**
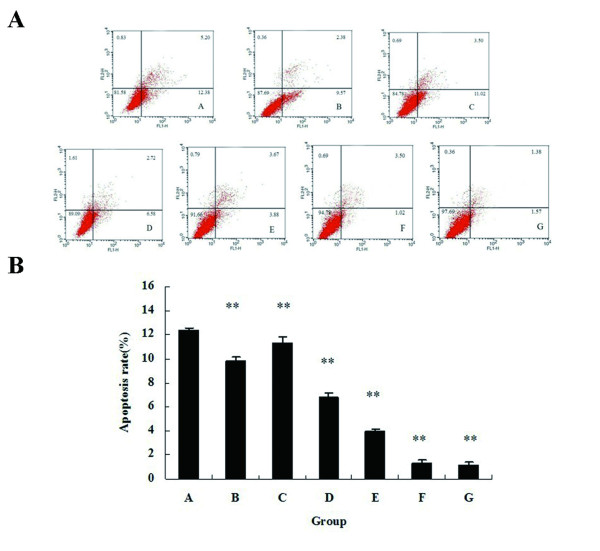
**Analysis of PC-3 cells apoptosis**. **A**. Flow cytometric detection of PC-3 cell apoptosis. The points of lower right quadrant represent apoptotic cells, and the points of upper right quadrant are on behalf of necrotic cells. The apoptosis rate of Group A was 12.38%, which was higher than those of the other groups. **B**. The average apoptosis rates of all 7 groups are shown in the bar chart. Higher percentage of apoptosis cells were detected by Annexin V-FITC and PI staining in Group A than in other groups. ** indicates P < 0.01 when compared to Group A.

Based on staining the cells with Annexin V-FITC and PI, flow cytometry was used to quantify the extent of apoptosis. The points of lower right quadrant in Figure 3A are on behalf of apoptotic cells. As demonstrated in Figure 3B, the maximal apoptosis rate of Group A was 12.51% (12.37 ± 0.17), only 80% of the living cells remaining, which was significantly higher than the other groups (P < 0.01). The apoptosis rates of Group F and G were much lower than other groups (P < 0.01), the highest apoptosis rate in Group F was only 1.57% (1.37 ± 0.18) and that of Group G was only 1.44% (1.14 ± 0.25), with no difference between these two groups (P > 0.05). The cell apoptosis rates of Group B-D were significantly higher than those in Group E to G (P < 0.01). And the cell apoptosis rate of Group E was higher than those of Group F and G (P < 0.01).

### Double-enhanced suicide gene system inhibits PC-3 tumor growth in vivo

**Figure 4:**
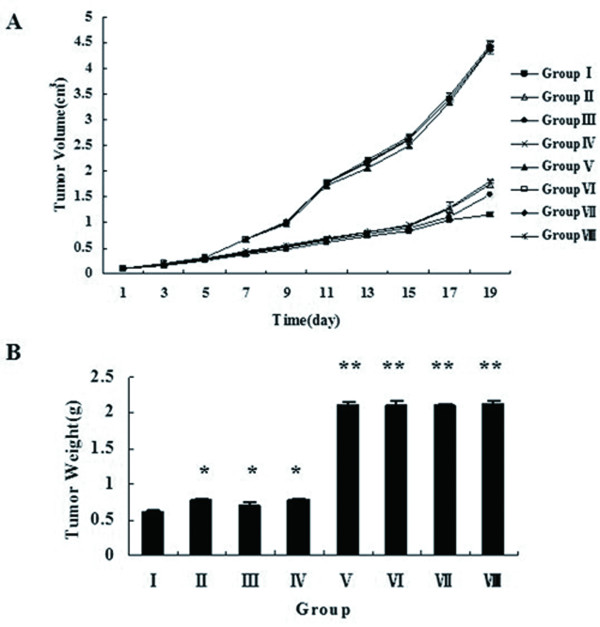
**Inhibition of tumor growth in nude mice model**. **A**. Tumor volumes were measured every 2 days after the first injection. The average calculated tumor volumes are shown for the indicated time points. The average tumor volumes of Group I to IV were smaller than other four groups (P < 0.01). The average tumor volume of Group I was the smallest than other groups (P < 0.01). No significant difference in average tumor volumes of Group V to VIII was seen during the whole procedure (P > 0.05). **B**. The bar chart shows the average tumor weights of all 8 groups. The average tumor weight of Group I was the lightest. * indicates P < 0.05, ** indicates P < 0.01 when compared to Group I.

**Figure 5:**
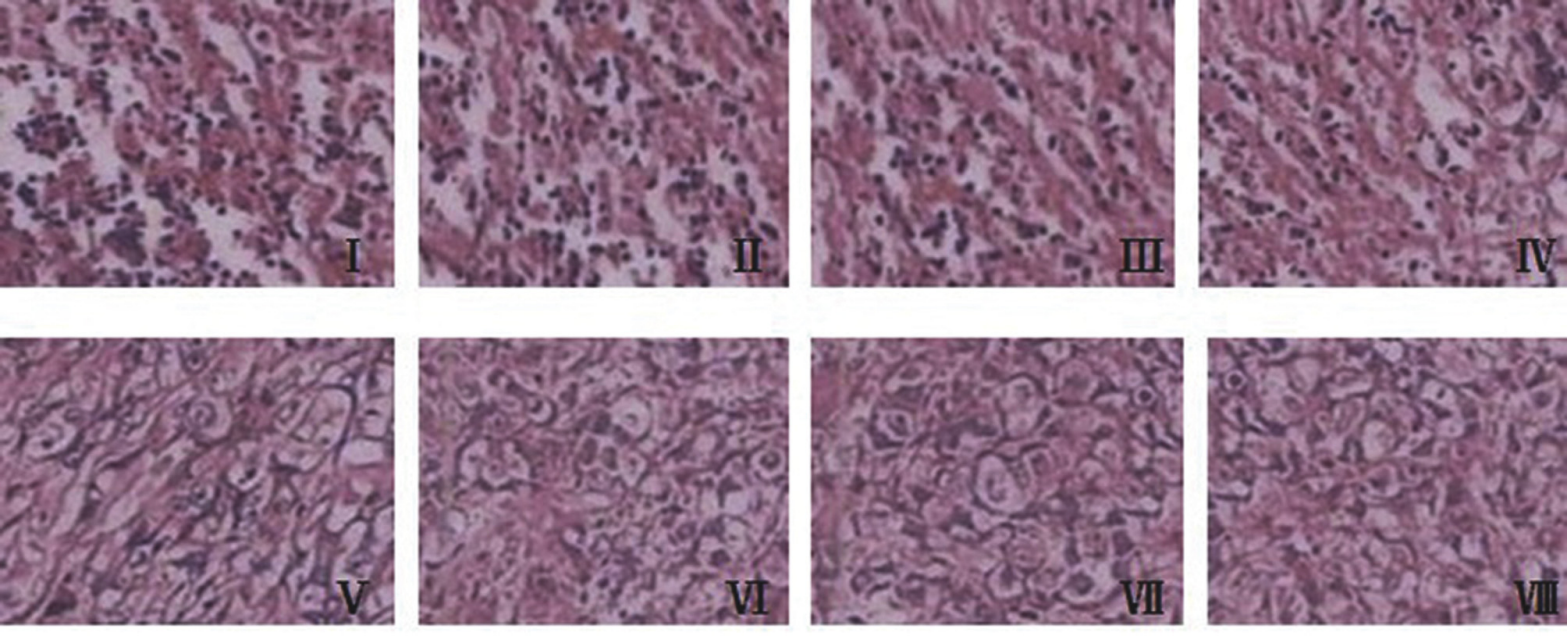
**Histological analysis of tumor specimen**. Hematoxylin and eosin staining (magnification × 100) in excised tumor tissues were conducted after treatment with different mixtures by intratumoral injection. Cell necrosis and hemorrhage with leukocyte infiltration in the tumors were observed from Group I to IV. No obvious pathological changes were shown from Group V to VIII.

To evaluate the effects of the suicide gene system on the PC-3 tumor growth in animal mode, the tumor volume and tumor weight were measured. Average tumor volumes were similar in all mice before therapy. All mice survived during the period of treatment. We found from Figure 4A that average tumor volumes of all groups had no significant difference during the first two days after treatment (P > 0.05). The average tumor volumes of Group I to IV were significantly smaller than other four groups (P < 0.01), and that of Group I was the smallest (P < 0.01). The average tumor volume of Group I reduced by about 70% after initiation of treatment compared with Group VIII. Average tumor volumes of Group V to VIII had no difference during the whole procedure (P > 0.05). As shown in Figure 4B, the final tumor weights of Group I to IV were significantly decreased than those of Group V to VIII (P < 0.01), and there was significant difference among these four groups comparing with each other (P < 0.05). The average tumor weight of Group I was the lightest in all groups (P < 0.01). The final tumor weight had no difference in Group V to VIII (P > 0.05). Specimens histological analysis was performed after tumors were isolated. The results shown in Figure 5 illustrated that there were extensive areas of coagulation necrosis and hemorrhage with leukocyte infiltration in the tumors from Group I to IV. On the other hand, any significant necrotic and hemorrhagic areas or leukocyte infiltration were not found in Group V to VIII.

## Discussion

One of the most frequently used and efficient suicide gene therapy approaches in cancer treatment is HSV-TK/GCV system [14]. There were a lot of researches about the HSV-TK/GCV suicide gene therapy of prostate cancer [11,15,16]. We have illustrated that HSV-TK/GCV system played a role in inhibiting the proliferation of PC-3 cells in vitro and in vivo. However, the primary problem that restricted the application of suicide gene therapy for prostate cancer is lack of suitable vector. Nowadays the studies about non-viral vectors are gradually increasing, such as liposomes, chitosan, polyethylene imine (PEI), polylysine (PLL), etc. PAMAM-D has its unique physical and chemical characteristics and advantages, which can provide favorable conditions for its application [13,17,18]. In our study, successful expression of TK and Cx43 in PC-3 cells transfected via G5-PAMAM-D was confirmed by RT-PCR and Western blot. This result indicated that G5-PAMAM-D as a gene vector could deliver recombinant plasmids into PC-3 cells successfully.

An important element of HSV-TK/GCV gene therapy was the"bystander effect", by which a high percentage of tumor cell death can occur, even when a low percentage of cells was transfected [19]. Suicide gene therapy became more effective when"bystander effect"enhanced [10]. Furthermore, it is believed that gap junctional intercellular communication via connexin (Cx) played a vital role in the "bystander effect" mediated by HSV-TK/GCV system [20]. Cx gene was considered as a non-mutant tumor suppressor gene, and it played a negative regulation role in the growth of most tumor cells including prostate cancer cells [21]. Cx43 is a gap junction protein containing 378 amino acids and its molecular weight is 43 kDa. Tumor progression and metastasis are correlated with reduction or absence of Cx43 gene expression [22]. It is reported that down-regulated expression of connexin play an important role in carcinogenesis and the "bystander effect" mediated by HSV-TK/GCV gene therapy [20]. Gemcitabine is a kind of deoxycytidine analogue and it can kill tumor cells by blocking DNA synthesis in tumor cells. Some scholars used gene therapy combining with gemcitabine for pancreatic cancer and glioma, and found the"bystander effect"significantly enhanced [23,24]. In our experiment, PC-3 cell growth was inhibited in Cx43-expressing cells comparing with the cells transfected with no Cx43. These results illustrated Cx43 played a role in enhancing the"bystander effect"and inhibiting cell proliferation. On the other hand, the cell growth inhibition rates and apoptosis rates in the cells treated with gemcitabine were significantly higher. These results illustrated that gemcitabine enhanced the"bystander effect"and inhibited PC-3 cells growth.

In line with the studies in vitro, we established prostate cancer nude mice model with PC-3 cells. After different experimental intervention, the status of tumor growth was observed. The results demonstrated that tumor growth was significantly inhibited by double-enhanced suicide gene system. Histological analysis showed that tumor tissues were damaged after administration of treatment system in our study visually. These experimental results in vivo suggested the system played a significant role of suppressing tumor progression.

In a word, our study implied that expression of Cx43 and gemcitabine could enhance the "bystander effect" of HSV-TK/GCV gene therapy. This double-enhanced suicide gene system was effective in inducing cell growth inhibition and apoptosis in vitro and suppressing tumor growth in vivo. It may be a potential approach for gene therapy of prostate cancer and could provide a choice to attempt suicide gene therapy for locally advanced or metastatic prostate cancer. Nonetheless, the effect of this therapy system on prostate cancer needs to be verified in other prostate cancer cell lines. In addition, we will also investigate the inhibitory effects of this system on tumor model in situ. This system will stand a good chance to be used for improving prostate cancer treatment combined with other therapies.

## Conclusions

This study illustrates that this new suicide gene system mediated by G5-PAMAM-D is effective in decreasing PC-3 cell proliferation and inducing cell apoptosis, and inhibiting tumor growth in vivo.

## Competing interests

The authors declare that they have no competing interests.

## Authors' contributions

YC carried out the experiments, analyzed the data and drafted the manuscript. GW conducted the experiments and participated in the design of the study. DK, ZZ, KY and RL assisted with experiments and help to draft the manuscript. WZ conceived of the study, and participated in its design. YX conceived of the study, participated in its design and revised the manuscript. All authors read and approved the final manuscript.
